# Supplementary material for: Crystal and melt inclusion timescales reveal the evolution of magma migration before eruption
Source: Nat Commun. 2018 Jul 9;9:2657. doi: 10.1038/s41467-018-05086-8 (PMC6037702; doi:10.1038/s41467-018-05086-8)
Supplement: Supplementary file 1 — Supplementary Information [file 41467_2018_5086_MOESM1_ESM.pdf]

Ruth et al. - Crystal and melt inclusion timescales reveal the evolution of magma migration before eruption

Supplementary Figure 1

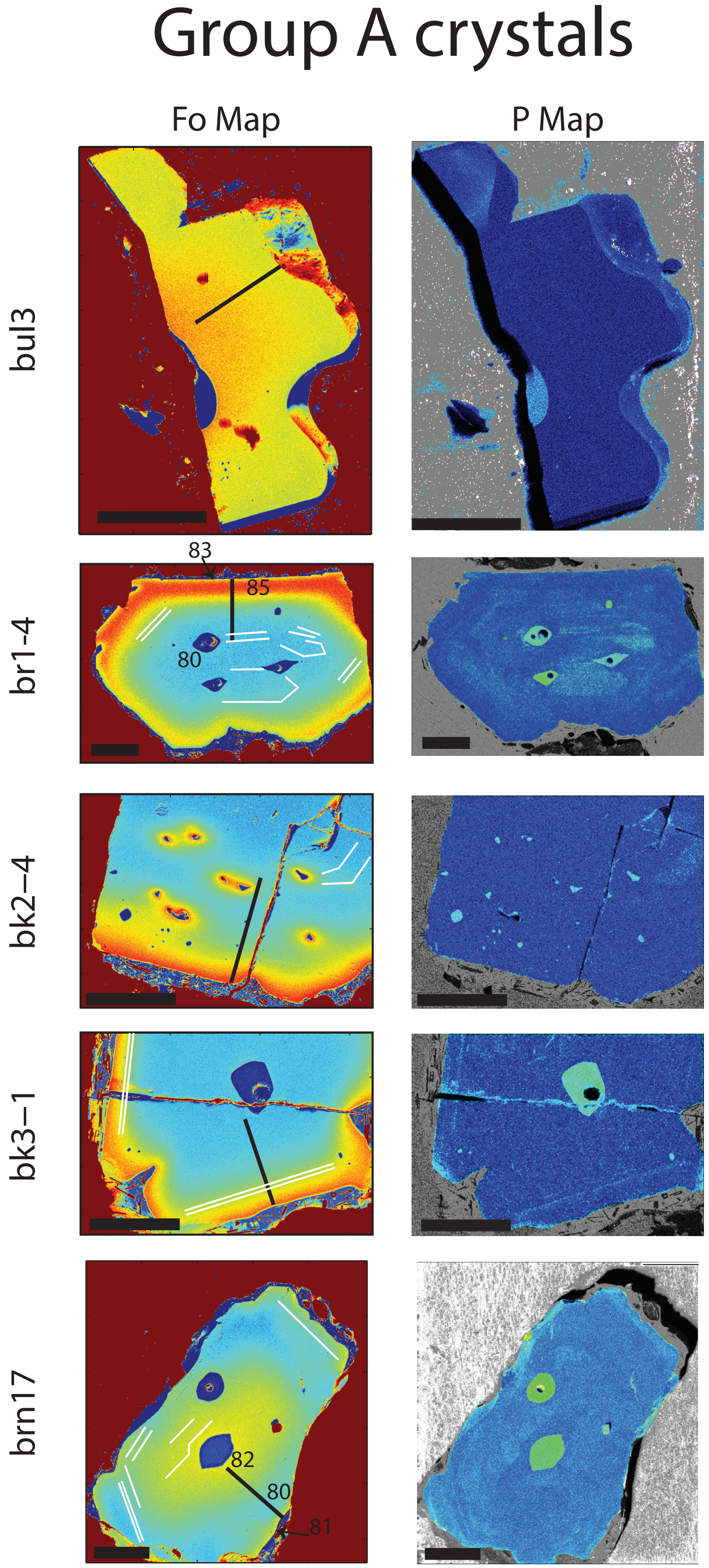

Supplementary Figure 1, continued

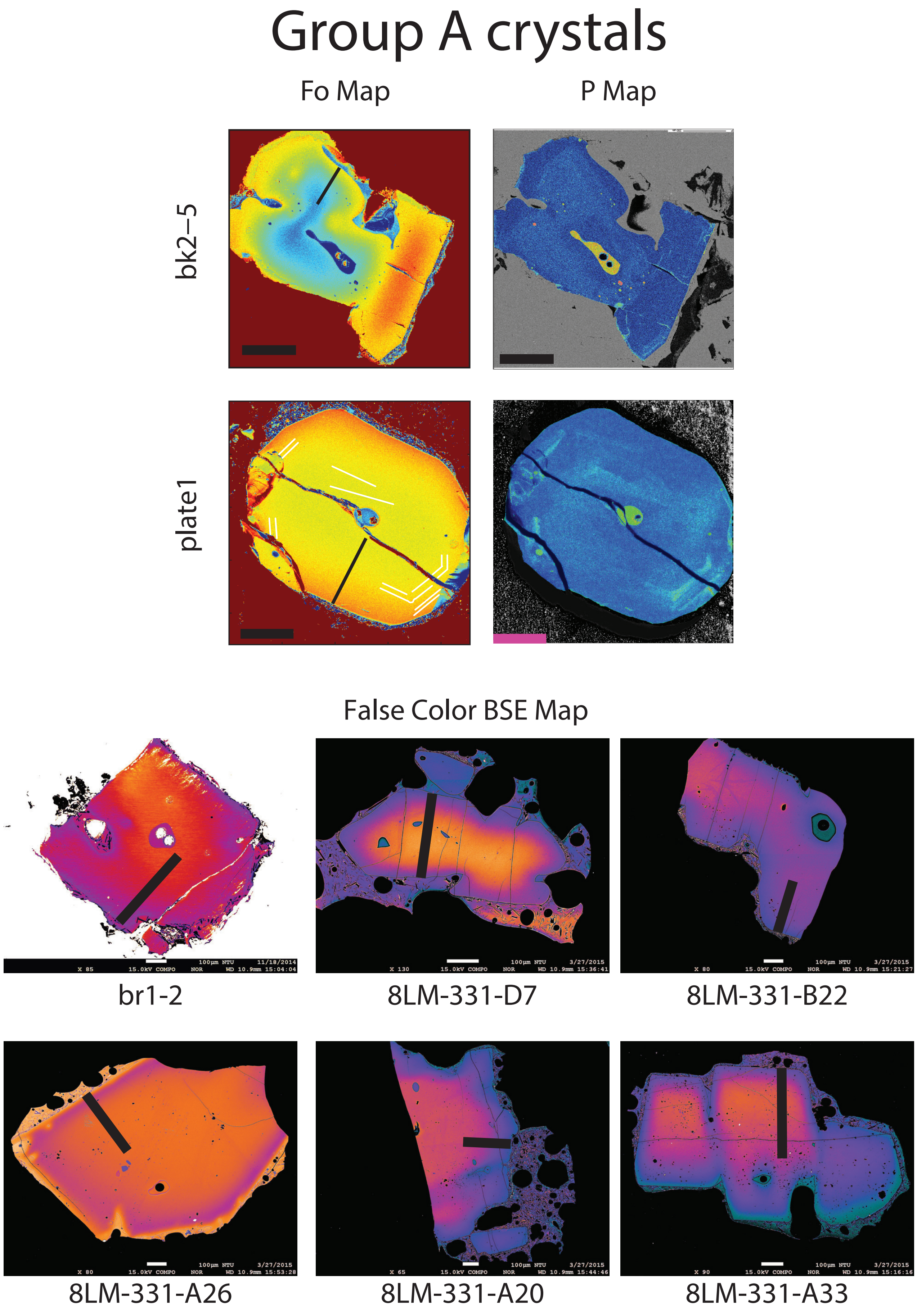

Supplementary Figure 1, continued

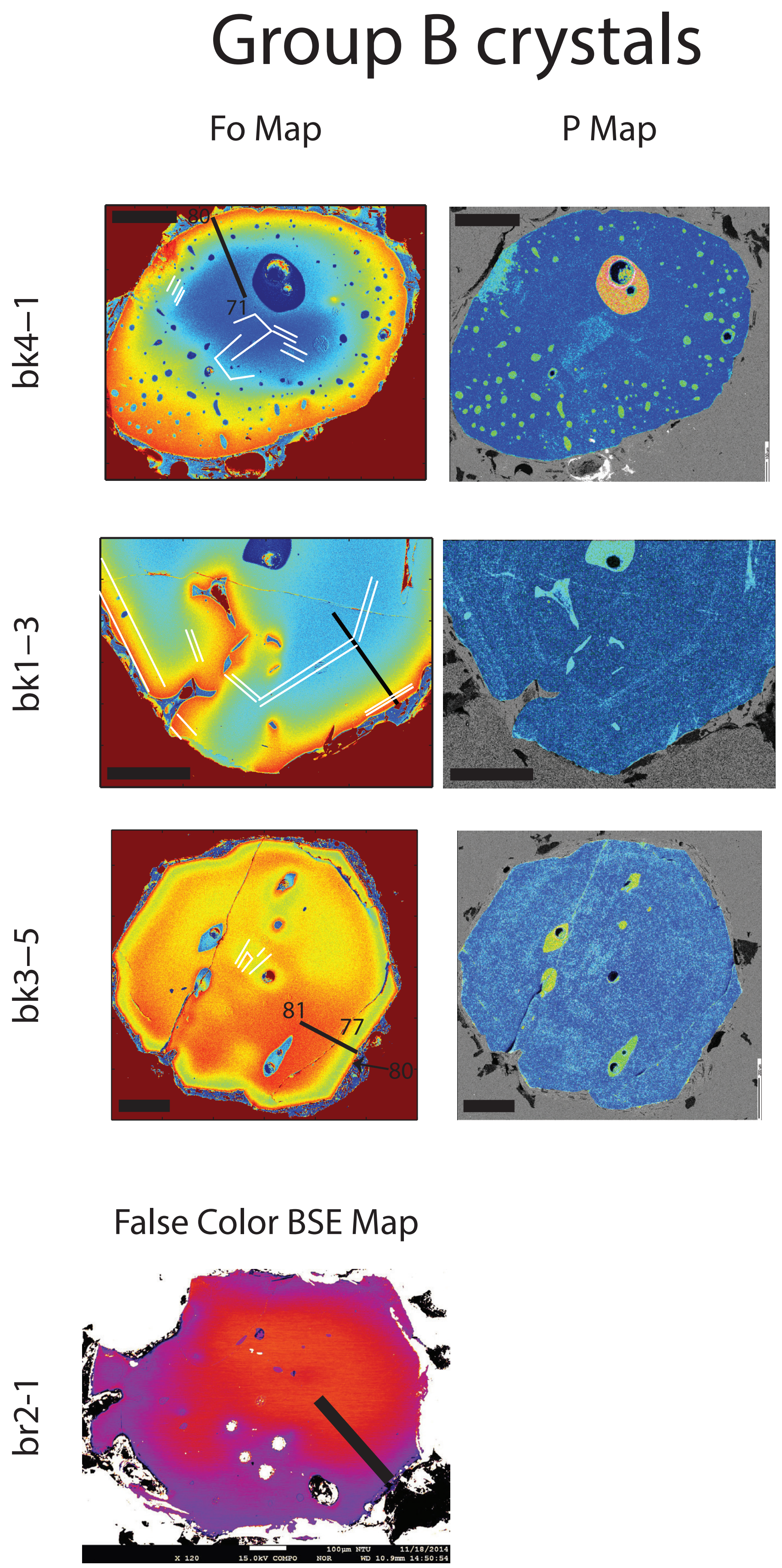

Group C crystals

Fo Maps P Maps

br5-1

br1-3

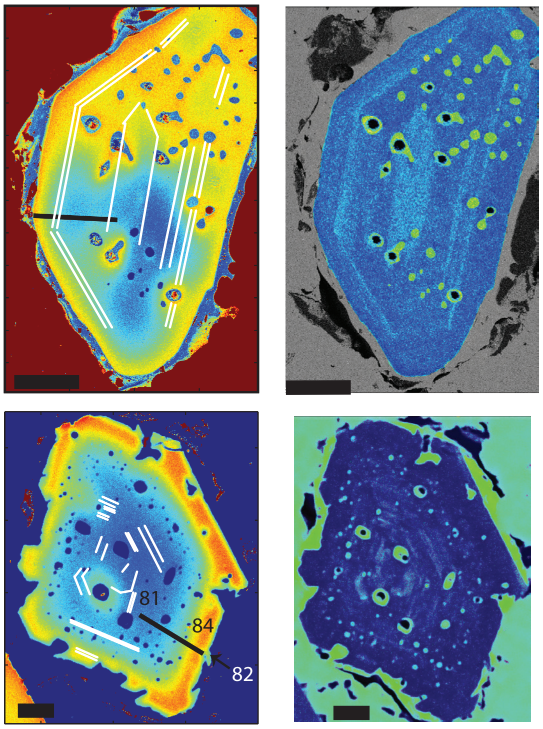

False color BSE Maps

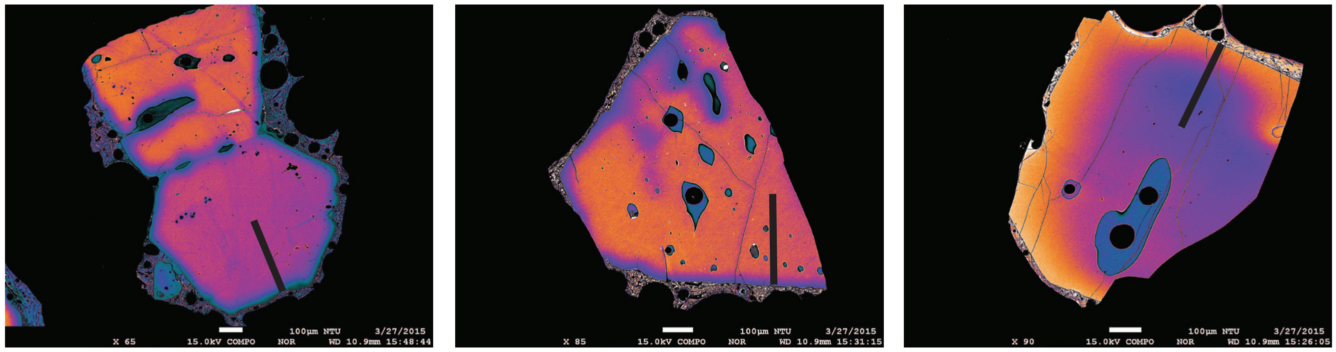

8LM-331A-A21 8LM-331A-D8 8LM-331A-C15

Supplementary Figure 1

Two dimensional zoning behavior for all crystals investigated in this study

Sample names are provided either at the side or beneath the respective images.

Semi-quantitative X-ray maps showing Fo and P content for selected crystals. Warm colors indicate relatively higher %Fo contents, where %Fo = 100 × Mg/(Mg+Fe). Traverse locations are shown with the back lines. Numbers adjacent to the black lines are quantiative %Fo contents of the respective crystals. White bars on the Fo maps show the regions of elevated P as ascertained from the P maps.

Scale bars are 200 µm unless otherwise noted. All samples beginning with 8LM, bul3, plate1, and brn17 were imbedded in epoxy. The rest were imbedded in indium.

False color backscattered electron images are provided for the rest of the sample suite. Warm colors indicate relatively *lower* %Fo contents. For example 8LM-331A-C15 is normally zoned whereas 8LM-331A-D8 is reverse zoned.

Supplementary Figure 2

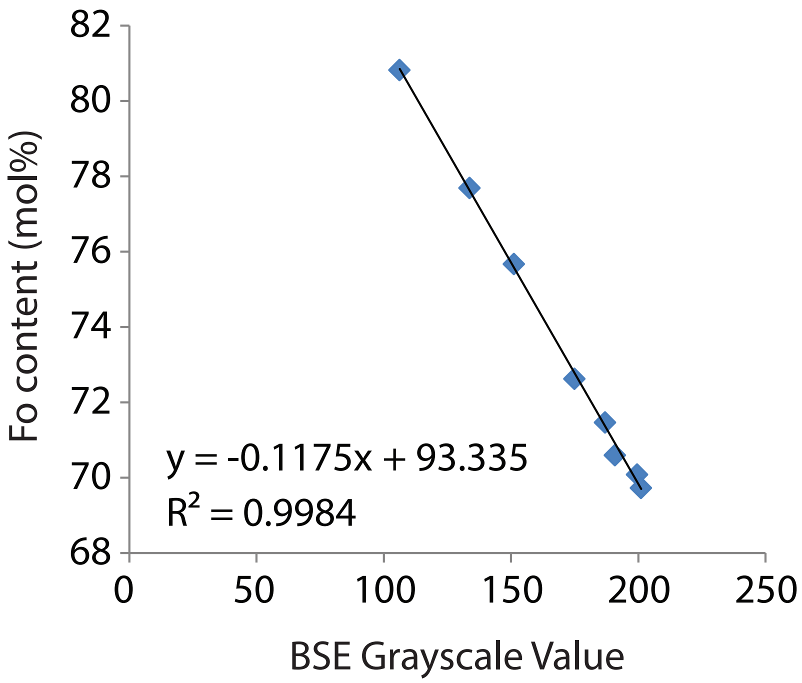

Supplementary Figure 2

Calibration curve for calibrated backscattered electron images

Fo contents were measured using electron microprobe analytical techniques outlined in the main text. Grayscale values were measured using techniques also outlined in the text.
