# Supplementary material for: Crystal and melt inclusion timescales reveal the evolution of magma migration before eruption
Source: Nat Commun. 2018 Jul 9;9:2657. doi: 10.1038/s41467-018-05086-8 (PMC6037702; doi:10.1038/s41467-018-05086-8)
Supplement: Supplementary file 3 — Description of Additional Supplementary Files [file 41467_2018_5086_MOESM3_ESM.pdf]

Description of Additional Supplementary Files

File Name: Supplementary Data 1
Description: Crystal classification scheme

File Name: Supplementary Data 2
Description: Complete electron microprobe dataset for all traverses. Note we have submitted this for long-term storage to the EarthChem data repository. The table is called out in the main text as Supplementary Data 2, cited in the references (Results section, crystal classification and chemistry - line 155), and called out in the Data Availability section.

File Name: Supplementary Data 3
Description: This has three main parts: 1) Model conditions and results for the DIPRA model runs. 2) Melt inclusion volatile contents, size, and location. 3) Compiled results table with diffusion timescale, pressure and volatile contents

File Name: Supplementary Data 4
Description: An excel file showing comparison for diffusion in different directions on same crystal.

File Name: Supplementary Data 5
Description: An excel file providing the 10 min and cumulative RSAM data used in this study.
